# The patterns and distribution of female genital tuberculosis among Togolese patients

**DOI:** 10.11604/pamj.2022.43.62.32965

**Published:** 2022-10-07

**Authors:** Toukilnan Djiwa, Panakinao Simgban, Yendoubé Kambote, Mayi Bombonne, Bagassam Mézéwè Sama, Baguilane Douaguibe, Mazamaesso Tchaou, Abdoul-Samadou Aboubakari, Tchin Darré

**Affiliations:** 1 Department of Pathology, University Teaching Hospital of Lomé, Lomé, Togo,; 2 Department Obstetrics and Gynecology, University Teaching Hospital of Lomé, Lomé, Togo,; 3 Department of Radiology, University Teaching Hospital of Lomé, Lomé, Togo

**Keywords:** Female GT, bleeding, inflammatory granuloma, caseous necrosis, Togo, Intertropical Africa

## Abstract

Tuberculosis is a real public health problem in developing countries. The aim of our article was to study the epidemiological, clinical, diagnostic characteristics of female genital tuberculosis in Togo. This was a descriptive and cross-sectional study on all cases concerning histologically diagnosed female genital tuberculosis in the department of pathological anatomy of Lomé in 1997-2018 (20 years). We collected 22 cases of women's Genital tuberculosis (GT), representing 2.2% (1008 cases) of extra-pulmonary tuberculosis. The mean age of the patients was 33.8 ± 0.2 years. Nine (9) patients had a history of treated GT. Depending on the location, the ovaries and fallopian tubes were the most affected (n=9 cases, 40.9%). Eighteen patients (81.8%) had at least one immunosuppression factor including HIV in 13 patients (72.2%). The reasons for consultation were metrorrhagia and pelvic pain with an associated mass in 7 women discovered on clinical examination and imaging. The macroscopic appearance of the specimens was suggestive of the diagnosis of genital tuberculosis in 12 cases (54.5%). Histology revealed caseous necrosis isolated in 3 patients (13.6%) and associated with gigantocellular epithelioid granulomas in 19 patients (86.4%). The patients received standard antibiotic treatment combining rifampicin, isoniazid, ethambutol and pyrazinamide. Genital tuberculosis is a rare extra-pulmonary location in Togo, often occurring in women with HIV, and the clinical polymorphism can lead to confusion with gynecological cancers.

## Introduction

Tuberculosis is of significant public health importance globally, with the largest number of diseases documented in sub-Saharan Africa [1]. The World Health Organization (WHO) estimated about 10.4 million tuberculosis cases with about 1.8 million deaths from TB in 2015 worldwide [2]. The lung remains the first organ affected by this endemic, although all viscera may be affected by this infection [3]. Female genital tuberculosis (GT) is one of the rare and little known forms of extra-pulmonary tuberculosis. This is an uncommon pathology in developed countries, affecting mainly women from disadvantaged backgrounds. In the majority of cases, the causative agent is *Mycobacterium tuberculosis* and more rarely *Mycobacterium bovis* [4]. It poses a problem of differential diagnosis with malignant tumors, in particular, because of clinical similarities [4,5]. Only an anatomopathological and/or bacteriological examination can confirm the diagnosis and make it possible to differentiate from a malignant disease [3]. The purpose of our work was to study the epidemiological, clinical, diagnostic characteristics of female genital tuberculosis in Togo.

## Methods

We carried out a descriptive study whose data were collected retrospectively in the laboratory of Pathological Anatomy of the University Hospital of Lomé in Togo. Togo is a small country of 56,600Km^2^, with an estimated population of 7,200,000 inhabitants, located between Ghana in the west and Benin in the east. We consulted the files of patients followed in Gynecology, from January 1997 to December 2018, for a period of 20 years, for whom a histological diagnosis of female genital tuberculosis was made by the Pathology Anatomy Laboratory of University Hospital Center of Lomé. These samples were previously fixed at 10% formalin. The different samples had undergone the usual paraffin embedding and cutting techniques followed by hematoxylin-eosin staining. We reviewed the results of HIV serology, the macroscopic and histological aspects of female genital tuberculosis lesions. The extra-pulmonary tuberculous sites were searched.

**Ethical approval and consent to participate:** this study received approval from the head of the laboratory department to be conducted. Since it was counting records, patient consent was not required. However during the counting and data collection patient names were not collected in order to preserve confidentiality. The manuscript has not been submitted to more than one review for simultaneous review and has not been published before. Only one study is not divided into several parts and no data has been produced.

**Consent for publication:** the Department of Pathology of Teaching Hospital of Lomé authorized the publication of this manuscript (Ref N° 18/2019/LAP/CHUSO).

**Availability of data and materials:** the datasets supporting the conclusions of this article are included within the manuscript and its supporting material.

**Funding:** the authors did not receive any funding from any source to carry out this study.

## Results

In total, we collected 22 cases of GT of the woman. During this same period, 1008 extra-pulmonary tuberculosis were diagnosed, a proportion of 2.2%. The mean age of the patients was 33.8 ± 0.2 years (11-64 years). Nine (09) patients had a history of treated tuberculosis: lymph node (n= 5) and pleural (n= 4). Depending on the location, the ovaries and the fallopian tubes were the most affected organs in 9 cases (40.9%) (Table 1). Tuberculin intradermal reaction in 5 patients was positive (≥10 mm) in 3 cases, and phlyctenular in 2 cases. Eighteen patients (81.8%) had at least one immunosuppression factor: an HIV infection was known in 13 patients (72.2%); four patients (22.2%) were hypertensive and one (5.6%) had a diabetic patient.

**Table 1:** the seats of tuberculosis lesions

	Number of cases (n)	%
Tubes and ovaries	9	40.91
Tubes	6	27.27
Ovaries	3	13.64
Endometrium	2	9.1
Tubes + Ovaries + endometrium	1	4.54
Cervix	1	4.54
Total	22	100.00

Twenty patients (90.9%) for whom we have information concerning their social conditions were: unemployed housewives (n=15 cases, 75%), farmers (n=3 cases, 15%) and pensioners (n=2 cases, 10%). Table 2 summarizes the epidemiological characteristics of the patients. For all the cases, the beginning was insidious. The average consultation time was 10 months and 6 days (range: 2 months-7 years). The clinical picture was polymorphic. One or more signs of tuberculous impregnation were reported in 13 patients. Weight loss was reported in 11 cases, anorexia in 10 cases, fever and asthenia in 9 cases each. The reasons for consultation were metrorrhagia and pelvic pain with an associated mass in 7 women discovered on clinical examination and imaging.

**Table 2:** epidemiological characteristics of patients

	Values
**Age (years)**	
Average	33.8±0.2
Extrems	11-64
**Profession**	
Unemployed	15/22
Farmers	3/22
Retirees	2/22
**Immunosuppressive factor (18/22)**	
HIV	13/18
Upper tension	4/18
Diabetic	1/18
**Antecedent of tuberculosis (9/22)**	
Ganglion	5/9
Pleural	4/9

The samples sent for histopathological analysis were for suspected cancers (n=17 cases, 77.3%) after clinical examination (n=7 cases) and on imaging techniques (n=10 cases). The samples examined consisted of biopsies (8 cases), total hysterectomy pieces with bilateral adnexectomy (10 cases) and without annexectomy in 4 cases. The macroscopic appearance of the specimens was suggestive of the diagnosis of genital tuberculosis in 12 cases (54.5%). Histology revealed caseous necrosis isolated in 3 patients (13.6%) and associated with gigantocellular epithelioid granulomas in 19 patients (86.4%). Chest X-ray was abnormal in 16 patients (72.7%), with pleurisy in 9 patients and in parenchymal lesions suggestive of tuberculosis in 4 patients. The patients received standard antibiotic treatment combining rifampicin, isoniazid, ethambutol and pyrazinamide for 2 months followed by a dual therapy with isoniazid and rifampicin for a total duration of 12 months. The Thirteen HIV/AIDS infected patients were on first line antiretroviral therapy (tenofovir/lamivudine /efavirenz) in ten patients and second line (tenofovir/lamivudine /atazanavir/ritonavir) in three patients.

## Discussion

Limitations of the study: the laboratory of Pathological Anatomy of the University Hospital of Lomé in Togo is the only public pathological anatomy laboratory in the country; therefore, our study could not be exhaustive because of the non-conveyed samples. Moreover, our study has all the limitations of retrospective studies, namely missing data, especially the age and the precise location of the lesions.

In developing countries such as ours, tuberculosis remains highly endemic. The extra-pulmonary form is experiencing renewed interest due to an unexplained increase in their relative frequency, reaching 20 to 40% depending on the series [2]. In particular, the female genital site remains underestimated and rarely cited in the literature, which explains the delay in diagnosis. The exact prevalence of female genital tuberculosis is difficult to specify because of many cases that are often asymptomatic [1,5]. The young age observed in the patients of our series is also reported in other African and Asian series, and could be explained by early marriages and pregnancies in this population [5]. In addition, the atrophic endometrium of elderly women is not a favorable environment for the development of *Mycobacterium tuberculosis* and this would explain the rarity of tuberculosis in postmenopausal patients [6]. HIV infection has increased the incidence of genital tuberculosis in India and Africa due to immunosuppression [7].

Genital tuberculosis often occurs in immunocompromised individuals and may be primary when no other GT focus is detectable or secondary, when a source can be identified, mainly pulmonary localization [8]. The patients of our series were of modest socio-economic condition with promiscuity. There is a significant correlation between socio-economic conditions and the prevalence of tuberculosis. Poor hygiene and promiscuity are recognized as risk factors favoring the emergence of tuberculosis [7,9]. No functional or physical sign is pathognomonic of GT; this pathology is characterized by a large clinical polymorphism [3,9]. Whereas classical ascitic forms and pelviperitonitis were the most common previously, the current forms are rather pauci-symptomatic and often fortuitous discovery, for example in the context of a primary or secondary infertility assessment [6]. Metrorrhagia and abdominal pain were the reasons for consultation when associated with a mass. In fact, patients with GT have deceptive clinical and radiological symptoms, such as ascites or abdominal distension, leading to the suspicion of malignant tumors, particularly in older patients [10]. In addition, GT is sometimes accidental discovery during investigations of infertile women [4]. Imaging is most often used in the tubo-ovarian masses [11]. Ultrasound shows tubo-vascular masses with calcification and fluid effusion in the Douglas [12]. The upper genital tract (fallopian tube and endometrium) are mainly affected by TG famine [13]. However, some studies have shown maximal endometrial involvement [2].

The diagnosis of GT famine is based on several diagnostic tools. Since the disease is usually silent with nonspecific and misleading signs, diagnosis can be difficult. Patients with tubo-vascular tuberculosis associated with peritoneal involvement (ascites) and elevated serum CA-125 levels are often misdiagnosed as ovarian carcinoma and undergo unnecessary and aggressive surgery [11,14]. Our series shows a significant proportion of cases of suspected malignancy (77.3%). Tuberculosis genital lesions are often pauci-bacillary, explaining the low sensitivity of acid fast staining tests (Ziehl-Neelsen staining) or the culture of *Mycobacterium tuberculosis* [14]. Polymerase chain reaction (PCR) is very sensitive for the diagnosis of genital tuberculosis. Although rarely used, it is recommended in cases with negative culture results or for the differential diagnosis between other forms [15]. Histopathological analysis is a very useful diagnostic tool because GT female are usually paucibacillary. This diagnostic tool also plays a major role in the elimination of malignant tumors. Anatomopathological examination confirms the diagnosis when there is a tuberculoid granuloma or giganto-cellular granuloma associated with caseous necrosis or when the bacillus of Koch is found on the histological sections [16]. In our series, the diagnosis was confirmed histologically in 100% of cases.

The treatment is primarily medical antibiotic therapy combining isoniazid, rifampicin, pyrazinamide and ethambutol, with regular clinical and paraclinical [17]. Complementary surgery is justified only in the presence of large lesions that respond little or not to antituberculous treatment or to a desire for pregnancy [18]. Indeed, according to the guidelines for HIV/AIDS prevention, antiretroviral therapy is recommended for all people with HIV/AIDS as soon as possible, but after the diagnosis of tuberculosis, individuals should initiate antiretroviral therapy in two to eight weeks to avoid interactions between antiretroviral and anti-tuberculosis treatments and the risk of immune hyper activation [19]. Nevirapine is not often recommended for tuberculosis-HIV co-infection, because of its hepatotoxicity and which potentiates the hepatotoxic effect of anti-tuberculosis drugs [19].

## Conclusion

Genital tuberculosis is a rare extra-pulmonary localization, the actual incidence of which is probably underestimated in Togo. Genital tuberculosis often occurs in people who are immunocompromised to HIV, and of modest socioeconomic condition with promiscuity. Tuberculosis-HIV co-infection is associated with severe forms of tuberculosis and an increase in mortality in tuberculosis patients, hence the interest in strengthening joint activities to combat this co-infection. The polymorphism of clinical symptoms damaged by weight loss, asthenia, fever and bleeding, can sometimes suggest gynecological cancers. Histological examination gives the diagnosis of certainty by showing the specific lesions of tuberculosis and eliminates a cancerous lesion, the main differential diagnosis.

### What is known about this topic

- Genital tuberculosis is a rare extra-pulmonary localization;
- This is an uncommon pathology in developed countries, affecting mainly women from disadvantaged backgrounds;
- In the majority of cases, the causative agent is Mycobacterium tuberculosis and more rarely Mycobacterium bovis.

### What this study adds

- Underlines the importance of this pathology in our context;
- Problem of differential diagnosis with female genital cancers;
- Difficulty of adequate management.
